# *Notes from the Field*: Postflooding Leptospirosis — Louisiana, 2016

**DOI:** 10.15585/mmwr.mm6642a9

**Published:** 2017-10-27

**Authors:** Alean A. Frawley, Ilana J. Schafer, Renee Galloway, Aileen Artus, Raoult C. Ratard

**Affiliations:** ^1^Infectious Disease Epidemiology Section, Louisiana Department of Health; ^2^Epidemic Intelligence Service, CDC; ^3^Division of High-Consequence Pathogens and Pathology, National Center for Emerging and Zoonotic Infectious Diseases, CDC.

In August 2016, extensive flooding occurred in south-central Louisiana. Approximately 1 month after the flood, the Louisiana Office of Public Health received notification through electronic laboratory reporting of two patients with serologic evidence of leptospirosis (immunoglobulin M antibodies to *Leptospira* species). Both patients were hospitalized with severe illness at the time of laboratory testing and recovered after appropriate treatment. Hospital record review revealed that both patients were exposed to floodwater before illness onset. Because these two (sentinel) patients with leptospirosis represented a marked increase over the three cases reported in their respective parishes of residence during the previous 28 years (*1*), an investigation was undertaken to identify other cases of leptospirosis related to the 2016 flood.

Leptospirosis is a bacterial disease caused by infection with pathogenic *Leptospira* species *(**2**)*. Humans can be infected through direct contact with urine from an infected animal or by contact with urine-contaminated soil or water, often during flooding (*3*). Approximately 90% of patients with leptospirosis experience a nonspecific, self-limited illness with symptoms of fever, chills, nausea, or headache (*2*). Pain in the calf and low back muscles and conjunctival suffusion without purulent discharge are distinctive features (*2*). Approximately 10% of patients develop severe illness, which is characterized by any combination of jaundice, renal failure, aseptic meningitis, cardiac arrhythmia, gastrointestinal symptoms, pulmonary hemorrhage, or circulatory collapse and is associated with a 5%–15% case fatality rate (*2*).

Suspected leptospirosis cases were defined as the occurrence of fever with at least two nonspecific symptoms (myalgia, headache, jaundice, conjunctival suffusion, or maculopapular or petechial rash), or at least one diagnosis indicating severe illness (aseptic meningitis, renal insufficiency, pulmonary complications, electrocardiogram abnormalities, gastrointestinal symptoms, hemorrhage, or jaundice with acute renal failure) during August 13–September 21, 2016 in a patient exposed to floodwater (*4*). The Louisiana Early Events Detection System (LEEDS), a statewide electronic syndromic surveillance system, was queried to identify patients treated in hospitals serving the flood region during August 13–September 21 who had signs, symptoms, or diagnoses compatible with leptospirosis. The dates were selected to include the flooding period (August 11–August 20) and a leptospirosis incubation period beginning 2 days after flooding started and continuing through 30 days after water recession (*2*). Hospital records of patients meeting the symptoms or diagnosis components of the case definition were reviewed; patients without fever or with laboratory evidence supporting an alternative diagnosis were eliminated. The remaining patients were interviewed to ascertain floodwater exposure; those with floodwater exposure provided whole blood and urine specimens for leptospirosis polymerase chain reaction (PCR) testing and a serum specimen for microscopic agglutination test (MAT) testing. MAT was also performed on serum from both sentinel patients. An acute urine specimen from one sentinel patient was tested by PCR. All laboratory testing was performed by CDC.

LEEDS queries yielded 69 patients warranting medical record review. After eliminating patients who did not meet the case definition based on medical record review, 13 of 18 patients who met the case definition were contacted for interview; among these, four reported floodwater exposure and submitted blood and urine specimens. MAT and PCR were negative for *Leptospira* spp. infection among all LEEDS-identified patients. Leptospirosis was confirmed by MAT in both sentinel patients; urine PCR identified *Leptospira kirschneri* DNA in one sentinel patient.

*Leptospira* species are prevalent among Louisiana wildlife. According to the Louisiana Department of Wildlife and Fisheries (LDWF), anti-*Leptospira* spp. seroprevalence in the Louisiana feral swine population was 71% in 2015 (Rusty Berry, DVM, LDWF, personal communication, November 9, 2016), which is markedly higher than the 26% estimated by the United States Department of Agriculture in 2012 (*5*). LDWF surveillance also identified a substantial increase in leptospirosis in the deer population, from an average seroprevalence of 7% during 2007–2012 to 42% during the 2015–2016 hunting season. (Rusty Berry, DVM, LDWF, personal communication, November 9, 2016 and July 6, 2017). 

No additional confirmed cases of postflooding leptospirosis were identified. Nonetheless, cases might have been missed because of flood-related access to care difficulties and patients not seeking medical care for less than severe illness. However, given the endemicity of *Leptospira* spp. among Louisiana wildlife, including documented *L. kirschneri* in feral swine isolates (*6*), and the two recent flood-related cases of leptospirosis, a high index of suspicion for leptospirosis among patients with compatible symptoms and exposure to untreated water is warranted, especially during flooding. Educating the public about leptospirosis prevention and clinicians about its clinical presentation might decrease the prevalence of severe disease by enabling early identification and treatment.
